# Effects of Presentation Rate and Attention on Auditory Discrimination: A Comparison of Long-Latency Auditory Evoked Potentials in School-Aged Children and Adults

**DOI:** 10.1371/journal.pone.0138160

**Published:** 2015-09-14

**Authors:** Naseem A. Choudhury, Jessica A. Parascando, April A. Benasich

**Affiliations:** 1 Psychology, SSHS, Ramapo College of New Jersey Mahwah, Mahwah, New Jersey, United States of America; 2 Center for Molecular & Behavioral Neuroscience, Rutgers University, Newark, New Jersey, United States of America; Birkbeck College, UNITED KINGDOM

## Abstract

Decoding human speech requires both perception and integration of brief, successive auditory stimuli that enter the central nervous system as well as the allocation of attention to language-relevant signals. This study assesses the role of attention on processing rapid transient stimuli in adults and children. Cortical responses (EEG/ERPs), specifically mismatch negativity (MMN) responses, to paired tones (standard 100–100Hz; deviant 100–300Hz) separated by a 300, 70 or 10ms silent gap (ISI) were recorded under Ignore and Attend conditions in 21 adults and 23 children (6–11 years old). In adults, an attention-related enhancement was found for all rate conditions and laterality effects (L>R) were observed. In children, 2 auditory discrimination-related peaks were identified from the difference wave (deviant-standard): an early peak (eMMN) at about 100–300ms indexing sensory processing, and a later peak (LDN), at about 400–600ms, thought to reflect reorientation to the deviant stimuli or “second-look” processing. Results revealed differing patterns of activation and attention modulation for the eMMN in children as compared to the MMN in adults: The eMMN had a more frontal topography as compared to adults and attention played a significantly greater role in childrens’ rate processing. The pattern of findings for the LDN was consistent with hypothesized mechanisms related to further processing of complex stimuli. The differences between eMMN and LDN observed here support the premise that separate cognitive processes and mechanisms underlie these ERP peaks. These findings are the first to show that the eMMN and LDN differ under different temporal and attentional conditions, and that a more complete understanding of children’s responses to rapid successive auditory stimulation requires an examination of both peaks.

## Introduction: Mismatch Responses in School-Age Children and Adults

The auditory system is intricately involved in the temporal analysis of spoken language by facilitating the decomposition of an incoming sound stream into its constituent features [1–3]. Effective language acquisition is characterized largely by the ability of the auditory system to efficiently process the spatiotemporal properties of the acoustic signal. Studies have shown that the inability to accurately perceive, discriminate and decode the brief, rapidly successive spectrotemporal events (referred to here as rapid auditory processing, “RAP”) that constitute language is associated with developmental language disorders such as Specific Language Impairment (SLI) and dyslexia [4–6]; for reviews, see [1, 7–11]. It is also well established that auditory processing abilities in general, and RAP in particular, improve over the course of development [12–18]. What remains relatively unclear, however, is the role of active attention in facilitating RAP abilities at different developmental periods. In the present study we assess the effect of attention on RAP processing abilities, in adults (18–42 years) and school-aged children (6–11 years).

Studies of auditory processing frequently use the electrocortical mismatch response to assess neural responses to changes in the acoustic signal in adults, children [19, 20], and also in infants [21]. The mismatch negativity (MMN) is an event-related potential (ERP) component elicited by a deviant or rarely occurring stimulus presented within a stream of standard stimuli [22, 23]). The MMN occurs approximately 150–300 ms after the onset of a deviant stimulus, can be elicited passively and is thought to precede conscious awareness [22, 24]. The MMN component peaks earlier than attention-based components (approx. 150 ms from deviance detection in adults) and reflects the early sensory stages of sound processing. The MMN is a modality-specific component, with neural generators within auditory cortices [24]. The component is highly sensitive to acoustic stimulus properties and can be elicited when the acoustic difference is near psychophysical thresholds [25] or when stimulus differences are consciously imperceptible [26]. These properties make it ideal for the study of mechanisms underlying auditory perception.

The MMN originates bilaterally in the auditory cortex, with contributions from auditory association areas, medial geniculate nucleus of the thalamus, and hippocampus [22, 27–33]). The MMN and its characteristics have been extensively described in adults, however the literature describing the MMN’s developmental progression in childhood and the role of attention in its expression is not as extensive. Maturational changes in auditory ERPs include decreases in latency and amplitude as well as dynamic morphological changes [21, 34–35]. These maturational changes are thought to result from increased synaptic density in auditory cortex, increases in myelination as well as differences in the location and orientation of the mismatch generators [36–42]. Various studies have reported maturational changes in the latency and amplitude of the MM response. For example, using tone stimuli, the latency of an MM response has been shown to decrease between the ages of 3 to 44 months [43], 6 and 48 months [21], 4 to 10 years-of-age [35], and in 7 to 16-year-olds [44]. These findings are consistent with reports of latency differences in the MMN of adults to tones as compared to children [44]. In contrast, the MM response elicited by speech stimuli in school-age children and adults has been found to be similar in latency and morphology (i.e. in 5–10 yr olds, [45]: for just perceptibly different variants of /da/; [46]: for variants of /da/ and /ga/; [47]). Despite their apparent similarities, the adult and child MMN response may not be equivalent processes [48]. Indeed, differences in the *amplitude* of the mature and developing mismatch response have been documented with most studies reporting that the MMN is larger in children as compared to adults [44, 49–51].

Additionally, a second later “discrimination” component, the late difference negativity, (LDN) also with frontocentral topography, has been identified in children (for a review, see [52–56]). While the early MMN (eMMN) component occurs at about 150–400 ms after stimulus onset and is thought to reflect pre-attentive, automatic change detection processes associated with the adult MMN, the LDN occurs at about 400–600 ms [53, 57–59] and is hypothesized to reflect further processing (i.e. a second look) of the deviant stimulus, particularly when the stimuli are complex or near threshold. These findings suggest that the LDN may be non-modality specific, reflect integration of information from earlier processing stages [60] and possibly be dependent on the concomitant development of attentional mechanisms ([52, 56, 61]; for review see [62]). An LDN-like component has also been described in adults with complex stimuli [44, 63–65], but the amplitude of this component has been found to decrease rapidly with age [52], making it difficult to use in adult studies (but see findings from [63] on LDN and dyslexia, and Ortiz-Mantilla and colleagues, [66], on late and early bilinguals and LDN). In studies of children with language and reading difficulties, abnormal or absent responses have been reported for both the eMMN and LDN [61, 63, 67–71], supporting the idea that these two components are sensitive measures of language-related auditory discrimination.

While the MMN is thought to reflect a pre-attentive process, attention can influence the formation of the MMN. This can take place either by mediating the formation of the cortical representation of the standard stimuli, or by impacting the accuracy and resolution of the deviant stimulus representation [72]. For example, in typically developing populations the MMN amplitude is greater when subjects actively attend to stimuli that are difficult to discriminate [64, 73] as compared to easily discriminable stimuli [64, 74]. Attention-related enhancements of the MMN have also been found to differ between adults and children. In a frequency discrimination task, Gomes and colleagues [75] compared MMN amplitudes of 8–12 year-old children and adults, and showed that in adults the MMN responses were not significantly altered by attention, while in children the MMN to difficult frequency contrasts *were* modulated by attention. Sussman and Steinschneider [76] examined stream segregation abilities in adults and 9- to 12-year-old children using passively and actively acquired ERPs (MMN and P3b), as well as behavioral measures of discrimination and found that, in children, stream segregation perception abilities assessed behaviorally were reflected in ERPs acquired during active, but not passive, listening. In adults, however, ERPs in both conditions were similar, and these electrophysiological measures mirrored behavioral perception of stream segregation. The authors suggest that in 9- to 12-year-old children, attending to relevant acoustic input may enhance and refine auditory representations established during passive exposure, resulting in greater automaticity of auditory processing. These studies suggest that attention is a critical factor in the development of efficient, automatic auditory processing and in the short term, may drive plasticity, so that the neural response to a sensory event that is initially voluntary eventually becomes automatic. A major limitation of the existing literature however, is that, to date, nearly all of the studies assess the role of attention to adult MMN and/or the child eMMN but not the LDN.

Here, two experiments examine auditory processing using an oddball paradigm with temporally modulated non-speech stimuli. Mismatch negativity responses were recorded from adults and 6 to 11 year-old children in conditions where the participants were either required to attend or ignore (passive condition) the auditory signal. The goal of these studies was to characterize automatic auditory discrimination of temporally-modulated stimuli of varying difficulty in adults and children, and to investigate the comparative effect of attention on auditory processing in these two populations. Experiment 1 addresses this research question in adults while experiment 2 examines the same aims in children. In keeping with prior research it was hypothesized that MMN in adults, and the eMMN in children, elicited by easily discriminable stimuli (i.e., slower-rate stimuli in this study) would be more robust than those elicited by difficult-to-differentiate stimuli (i.e., fast rate) [22, 77]. It is also hypothesized that MMN and the eMMN would be enhanced by attention allocation to the stimulus, and finally that in children the LDN may be more variable (in amplitude or latency), particularly for the more difficult stimuli (fast rate) suggesting that these stimuli might engage mechanisms involved in “further”, more up-stream, acoustic processing.

## Methods: Effect of Attention and Rate on Auditory Processing in Adults

### Participants

Twenty-one monolingual English-speaking adults (12 males) with no history of hearing, neurological, or language/learning problems (including dyslexia, language delay or impairment, dyscalculia) were recruited to participate in this study (mean age = 27 years, range 18–42 years). All participants had completed high school and 12 (57%) had completed college (mean years of schooling post 8^th^ grade 8.11, SD 2.94). Rutgers University’s Institutional Review Board approved all study procedures and written informed consent was obtained from all participating adults.

### Stimuli

Stimuli were complex tones (70ms in duration) with a fundamental frequency of 100 or 300Hz including 15 harmonics (6 dB roll-off per octave). All stimuli were presented at 75 dB SPL free-field via speakers to the left and right of the participant. Tones were presented in pairs in a typical oddball paradigm using a blocked design with different interstimulus intervals (ISIs: the time between the 2 tones in a pair) of 300, 70, or 10ms. A trial consisted of the presentation of one tone pair and each block consisted of 833 trials. The standard tone pair was 100 –100Hz (85% probability: 708 trials), and the deviant tone pair was 100–300Hz (15% probability: 125 trials). Deviant tone-pairs were presented in a pseudo-random order with at least 3 and no more than 10 standards between each deviant. The ISIs were chosen based on previous research in our lab strongly suggesting that tone pairs with an interpolated 300ms ISI were easily discriminated, 70 ms ISI was of moderate difficulty and a 10 ms ISI was the most challenging to parse [66, 78–81]. Inter-trial intervals (ITI, onset to onset) were 700ms for the 300ms ISI block, and 705ms for the 70 and 10ms ISI blocks.

### Procedure

#### Study Design

All participants received two attention conditions, *Ignore* and *Attend*, and two rate-of-presentation conditions, 70ms ISI and 300ms ISI or 70ms ISI and 10ms ISI. The *Ignore* condition always preceded the *Attend* condition. Every participant received four blocks in the following order: *Ignore condition* with 70ms ISI tones, *Ignore condition* with either 300 or 10ms ISI tones, *Attend condition* with 70ms ISI tones, *Attend condition* with either 300 or 10ms ISI tones. This design minimized data loss due to participant fatigue. Pilot studies revealed that if all 6 blocks (2 attention and all 3 rate-of-presentation, 300ms, 70ms and 10ms ISI) were presented individuals became restless and their performance on the *Attend* conditions suffered significantly (as assessed by significant decreases in correct responses) [82]. Thus to maximize data quality and participant retention, each participant received two of the three rate conditions and therefore 2 groups were created based on rate-of-presentation. Participants in Group I were assessed on 70 and 300ms, (n = 12), and participants in Group II were assessed on 70 and 10ms (n = 9). This design also ensured that all participants received the 70ms ISI rate condition so that the two groups could be compared on rate processing equivalencies. As previously reported, Thomas et al., [82, 83] demonstrated that the MMN elicited by the 70ms ISI stimuli in Groups I and II did not differ in morphology and topography. These findings verify homogeneity of the MMN with the 70ms ISI stimuli between groups of adults and allow us to compare the responses to the 300 and 10ms ISI across group. During the *Ignore* condition, adults viewed a silent video with subtitles (n = 10) or read text (n = 11) and were asked to ignore the sounds. In the *Attend* condition, all participants were instructed to press a button located on a “response pad” resting on their lap as quickly as possible when they heard the deviant (target) tone pair (100Hz-300Hz). There were 33 training trials during which participants were given feedback. There was no feedback during the test sessions.

#### ERP Recording

Participants were seated in an acoustically shielded room. Geodesic Sensor Nets (GSN; Electrical Geodesics, Inc.) with 64 Ag/AgCl electrodes were used to measure EEG at all 64 sites. The vertex served as an online reference and vertical and horizontal eye movements (EOG) were measured with electrodes placed above, below and on the outer canthi of each eye. Online the EEG was sampled at 250 Hz with filters set at 0.1 to 100 Hz. Impedances were maintained below 50 kΩ.

#### Off-line EEG/ERP Analyses

All EEG data were processed using BESA (Brain Electrical Source Analysis V 5.1.8.10, MEGIS Software GmbH, 2006). The data were re-referenced off-line to an average (whole head) reference, and were scanned and corrected for lateral and horizontal eye movements [84–86]. The thresholds for EOG artifact correction were 150 μV for horizontal and 250 μV for vertical eye movements/eye blinks. Artifact rejection criteria were set to exclude activity exceeding 120 μV. A minimum of 50 artifact-free trials was averaged by stimulus type (deviant or pre-deviant standard), and baseline corrected (baseline = -100 ms). Data were then grand averaged for each participant and bandpass filtered at 1–15 Hz.

Individual grand-averaged difference waveforms (deviant minus pre-deviant standard) were visually inspected for the presence of MMN in the 100–150ms latency range following the onset of the deviant tone. This study focuses on MMN component in frontal, frontocentral and central channels in left and right hemispheres and hence MMN peak amplitude and latency values were extracted from the following channels: frontocentral (channel 17/Fc_3_, 54/Fc_4_), frontal (13/F_3_, 62/F_4_), central (21/C_5_, 53/C_6_,), temporal (24/T_7_, 52/T_8_), parietal (28/P_5_, 46/P_6_), and occipital (38/O_z_) channels (see [87] for verification of Geodesic sensor net electrode positions and their 10–10 international equivalents). Temporal channels were used to detect for the expected inversion of polarity seen for the MMN. As expected, topographic maps showed maximal amplitudes in frontocentral and central channels for the deviant as well as the difference waveforms and a relative inversion of polarity at mastoid channels. All MMN peak latencies presented here are relative to the onset of the second tone in the pair thus allowing for statistical comparison of latencies among different ISI conditions.

### Statistical Analyses

A 4-way Repeated Measures Analysis of Variance (2 x 2 x 3 x 2, ANOVA) was run in order to assess the effects of rate (2: *slow*, *fast*), attention (2: *Ignore*, *Attend*), regions of interest (ROI) (3: frontal, frontocentral, and central) and laterality (2: left, right) on the MMN peak. Given the nature of the study design, 2 separate analyses were conducted for Group I (G1), comparing performance on 300 and 70ms ISIs, and Group II (G2), comparing performance on 70 and 10ms ISIs. The 300 and 10ms ISI rates were compared using independent samples t-tests after verifying that Group I and Group II did not differ in amplitude or latency in the 70ms ISI.

## Results

### Morphology of Adult Waveform

**Fig 1:**
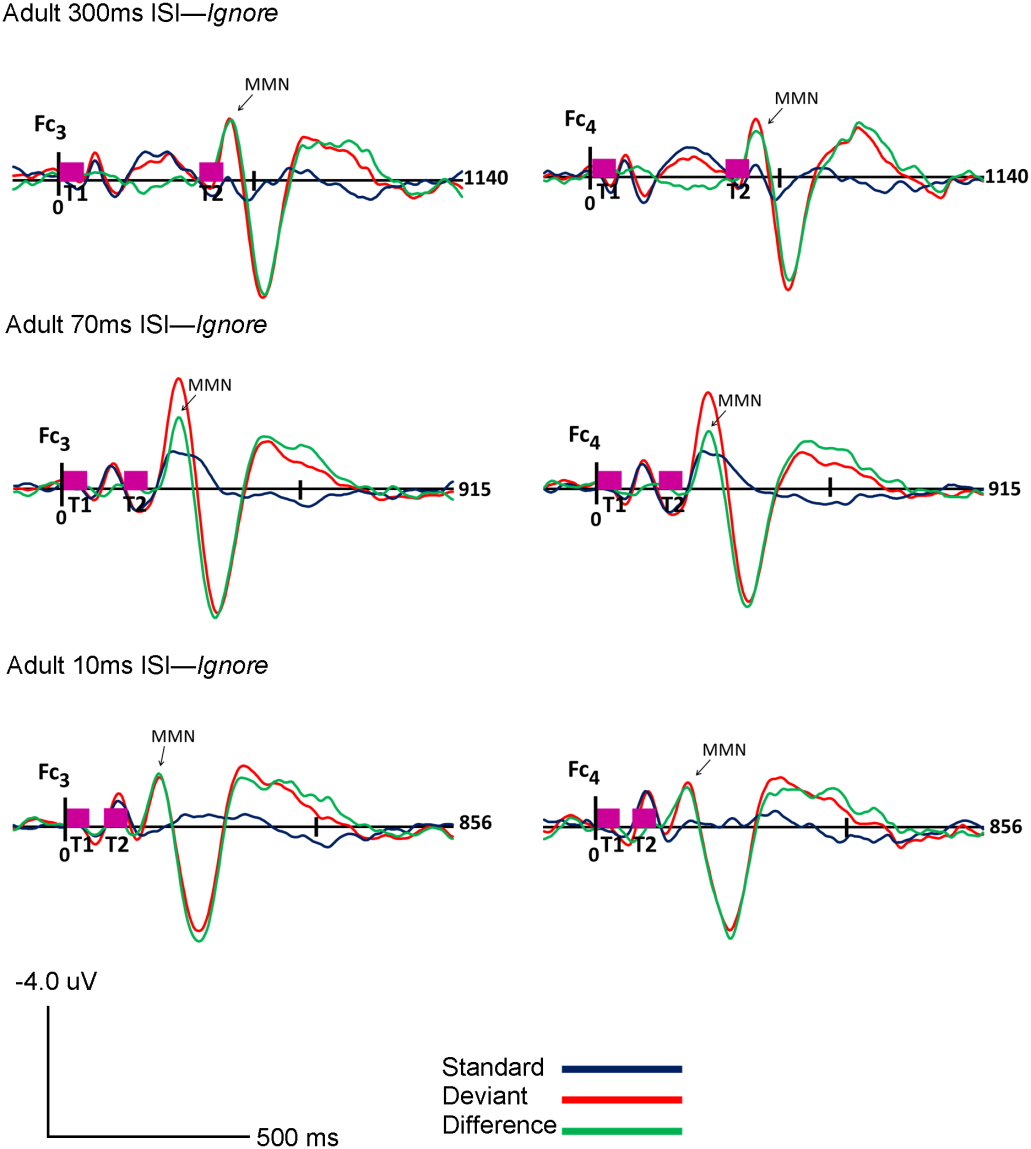
Adult *Ignore* Condition. Grand average ERP’s of the adults for the *Ignore* condition at Fc_3_ (Ch.17) and Fc_4_ (Ch.54) for 300ms (top), 70ms (middle) and 10ms (bottom) ISI’s are shown. Response to the standard stimuli (blue line), the deviant stimuli (red line) and the difference condition (standard- deviant; green line) are plotted. Vertical pink bars on the baseline indicate the onset of the tones. Tone 1 and tone 2 are paired stimuli. Negative voltages are plotted up, positive voltages are plotted down.

**Fig 2:**
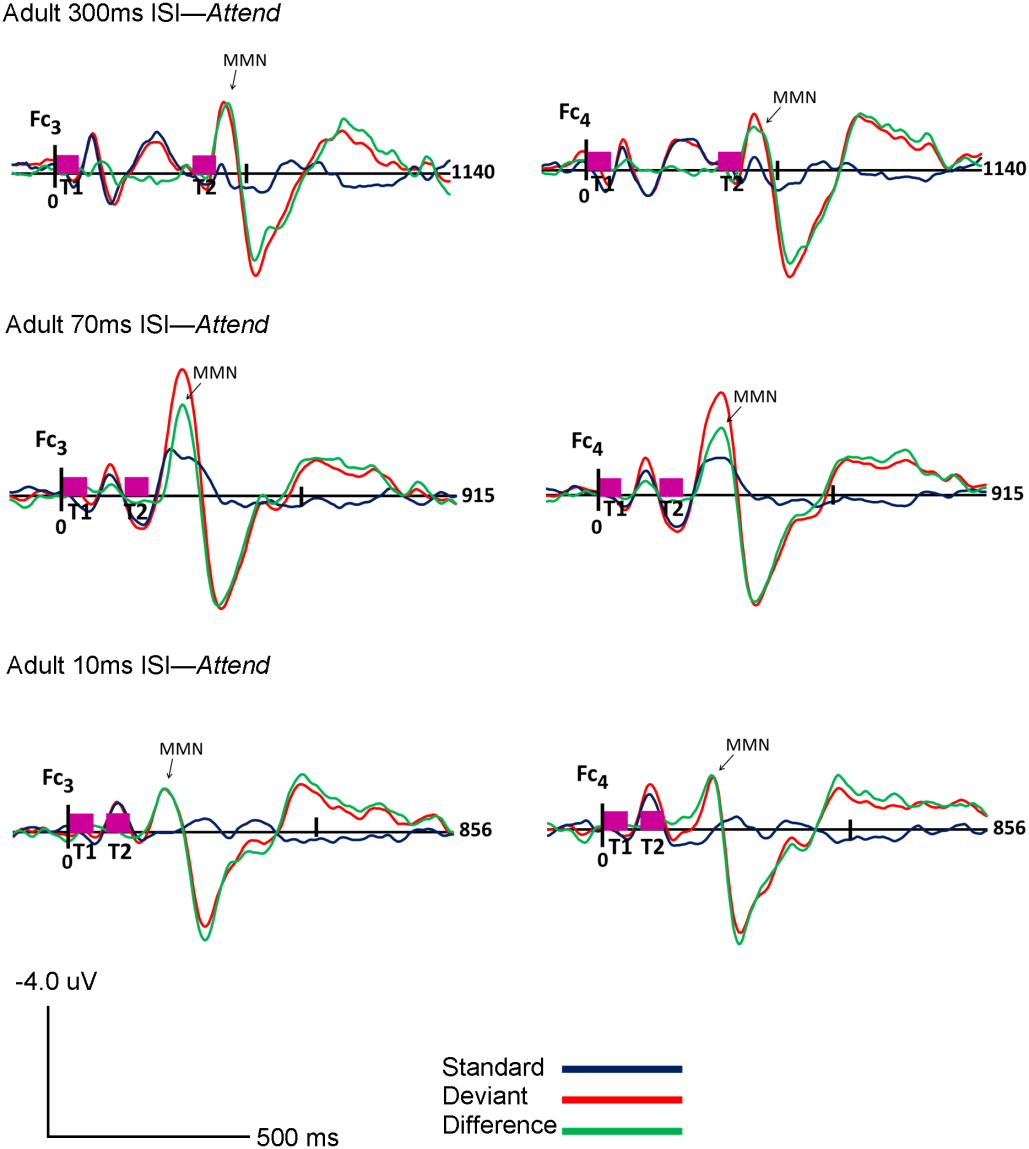
Adult *Attend* Condition. Grand average ERP’s of the adults for the *Attend* condition at Fc_3_ (Ch 17) and Fc_4_ (Ch 54) for 300ms (top), 70ms (middle) and 10ms (bottom) ISI’s are shown. Response to the standard stimuli (blue line), the deviant stimuli (red line) and the difference condition (standard- deviant; green line) are plotted. Vertical pink bars on the baseline indicate the onset of the tones. Tone 1 and tone 2 are paired stimuli. Negative voltages are plotted up, positive voltages are plotted down.

**Fig 3:**
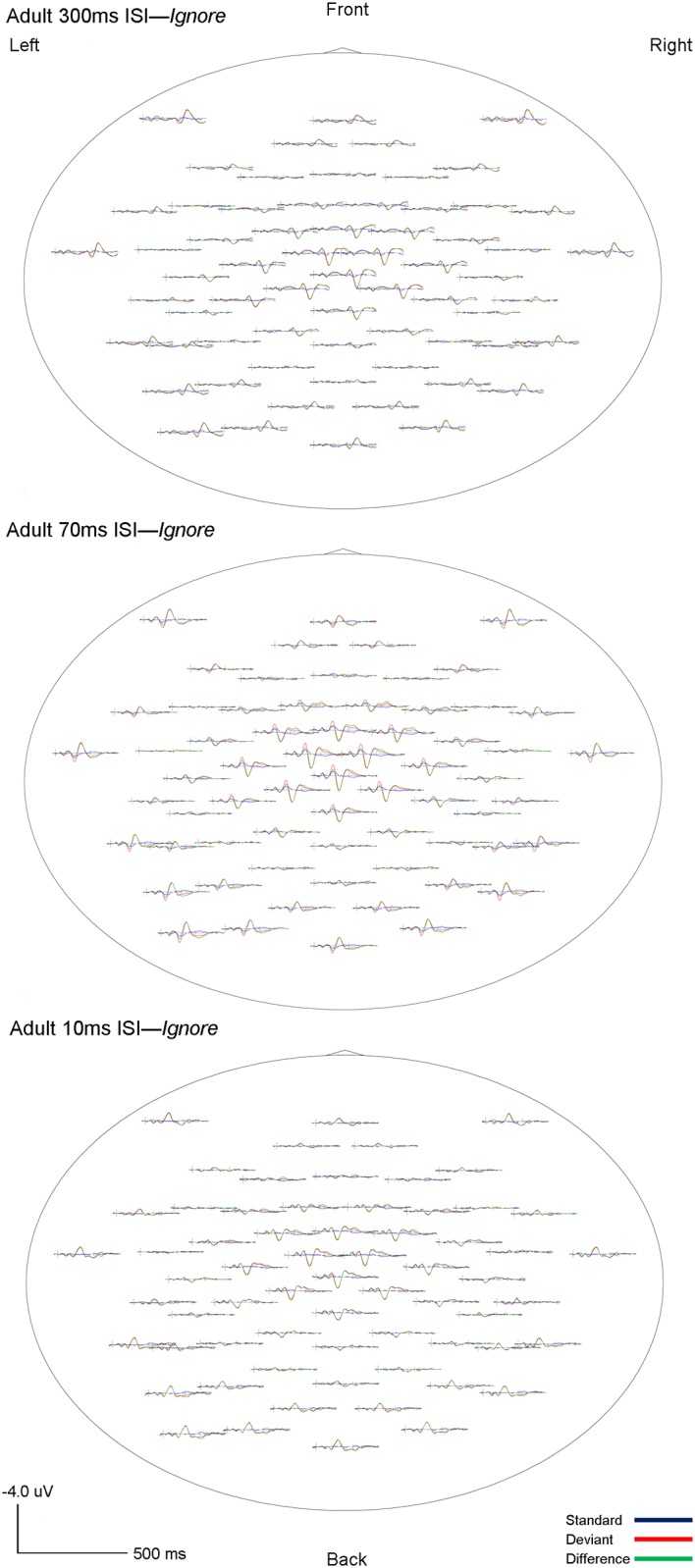
Adult *Ignore* grand averaged topographic maps. Whole-head topographies for grand average waveforms for the *Ignore* condition at Fc_3_ (Ch 17) and Fc_4_ (Ch 54) for 300ms (top), 70ms (middle) and 10ms (bottom) ISI’s are shown. Negativity up, positivity down. The standard wave is shown in blue, deviant in red, and difference (deviant-standard) in green. Vertical bars on the baseline indicate the onset of the tones. Tone 1 and tone 2 are paired stimuli.

**Fig 4:**
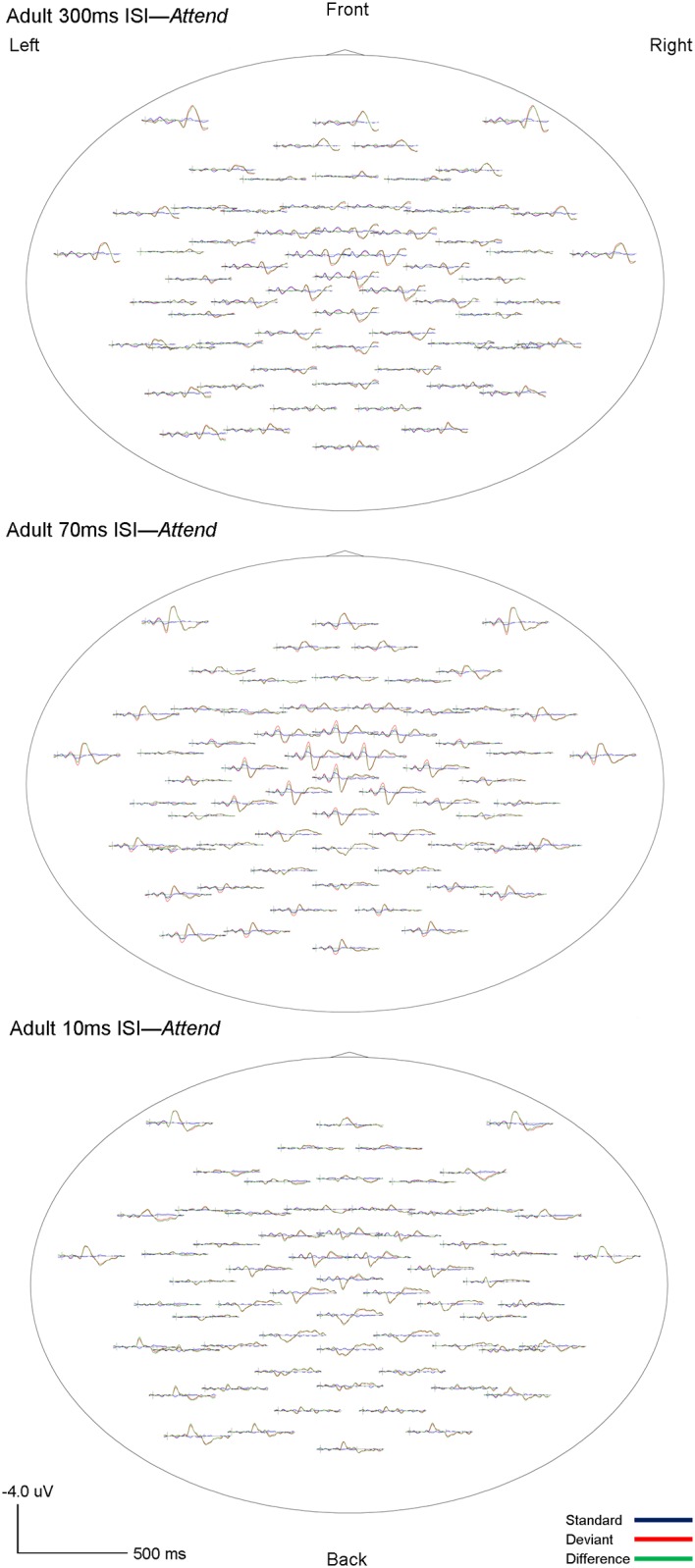
Adult *Attend* grand averaged topographic maps. Whole-head topographies for grand average waveforms for the *Attend* condition at Fc_3_ (Ch 17) and Fc_4_ (Ch 54) for 300ms (top), 70ms (middle) and 10ms (bottom) ISI’s are shown. Negativity up, positivity down. The standard wave is shown in blue, deviant in red, and difference (deviant-standard) in green. (deviant-standard) in green. Vertical bars on the baseline indicate the onset of the tones. Tone 1 and tone 2 are paired stimuli.

Visual inspection of the standard, deviant and difference grand-averaged waveforms revealed that adults displayed typical MMN morphology and topography. A MMN peak, maximal in frontocentral regions, between 110–160 ms was observed for all three rates (300, 70 and 10ms ISI) and in both the *Ignore* and *Attend* conditions (see Figs 1 and 2). Topography of the grand-averaged waveforms are shown in Figs 3 and 4

### Effect of Rate and Attention on Auditory Processing in Adults

#### Amplitude Effects

Results of the 4 way ANOVA revealed a Rate x Attention interaction (*G1*, *F* (1, 11) = 7.67, *p*<.05; *G2*, *F*(1, 8) = 6.82, *p*<.05). In both models, the more difficult-to discriminate rates (70ms and 10ms ISI) in the *Attend* condition elicited significantly larger MMN peaks compared to the *Ignore* condition, and in comparison to the 300 and 70 ms ISI’s (Table 1). A main effect for ROI and Attention was also found; *Frontocentral* channels had significantly larger peaks compared to *Frontal* and *Central* channels (*G1*, *F* (2, 10) = 11.36, *p*<.01; *G2*, *F* (2, 7) = 7.98, *p*<.01), and for both groups and all rates of presentation, attending to the stimuli lead to larger peak amplitudes.

**Table 1:** Means and Standard Deviations of the Mismatch Negativity (MMN) Amplitude (mV) in the *Ignore* and *Attend* condition for Adults.

	Group I: 300ms vs. 70ms (n = 12)				Group II: 70ms vs. 10ms ISI (n = 9)			
	300ms ISI Mean	SD	70ms ISI Mean	SD	70ms ISI Mean	SD	10ms ISI Mean	SD
*Location (ch)*	**Ignore Condition**							
Left Frontal (13)	-1.26	0.7	-1.40	0.6	-1.32	0.9	-1.27	0.8
Left Fronto-central (17)	-1.30	1.1	-1.51	1.0	-1.59	1.0	-2.16	1.2
Left Central (21)	-1.25	0.6	-1.25	0.7	-1.25	0.7	-1.18	0.5
Right Frontal (62)	-1.28	0.6	-1.65	0.9	-1.75	0.7	-1.54	0.8
Right Fronto-central (54)	-1.65	0.8	-1.88	0.9	-1.70	0.9	-1.99	0.9
Right Central (53)	-0.91	0.5	-1.19	0.6	-1.01	0.6	-1.12	0.5
	**Attend Condition**							
Left Frontal (13)	-1.56	0.7	-1.62	0.6	-1.74	0.7	-1.80	0.9
Left Fronto-central (17)	-2.25	1.2	-2.80	1.1	-2.75	1.4	-2.83	1.6
Left Central (21)	-1.39	0.8	-1.68	0.9	-1.51	0.6	-2.86	0.9
Right Frontal (62)	-1.55	0.5	-1.85	1.1	-1.91	1.9	-2.65	1.1
Right Fronto-central (54)	-2.24	0.8	-2.72	1.4	-2.79	1.6	-2.99	1.5
Right Central (53)	-1.24	0.6	-1.84	0.6	-1.81	0.6	-2.20	1.0

#### Latency Effects

Examination of latency differences revealed a main effect for Attention, *Ignore <Attend* (*G1*, *F* (1, 11) = 14.82, *p*<.01; *G2*, *F* (1, 8) = 16.02, *p*<.01,) and a main effect for Hemisphere, *Left <Right* (*G1*, *F* (1, 11) = 4.96, *p*<.05); *G2*, *F* (1, 8) = 5.47, *p*<.05,) for both groups. In addition for G1 only (300 versus 70 ms ISI) there was a main effect of Rate, *300ms < 70ms ISI* condition, (*G1*, *F* (1, 11) = 6.23, *p*<.05), and an independent sample t-tests comparing performance on 300 and 10ms ISI revealed significant latency differences for both the *Attend* and *Ignore* conditions on all frontal and frontocentral sites (*300ms < 10ms ISI* condition, *t*(20) = 2.2 to 3.6, *p*<.05). No significant Rate differences were observed between 70 and 10ms ISI conditions (Table 2).

**Table 2:** Means and Standard Deviations of the Mismatch Negativity (MMN) Latency (ms) in the *Ignore* and *Attend* condition for Adults.

	Group I: 300ms vs. 70ms (n = 12)				Group II: 70ms vs. 10ms ISI (n = 9)			
	300ms ISI Mean	SD	70ms ISI Mean	SD	70ms ISI Mean	SD	10ms ISI Mean	SD
*Location (ch)*	**Ignore Condition**							
Left Frontal (13)	100	22	120	9	119	9	126	29
Left Fronto-central (17)	112	21	121	6	124	6	119	21
Left Central (21)	111	21	128	20	127	18	117	24
Right Frontal (62)	100	33	117	4	116	6	115	25
Right Fronto-central (54)	112	19	117	7	117	7	116	23
Right Central (53)	115	22	127	20	135	25	127	31
	**Attend Condition**							
Left Frontal (13)	117	30	152	34	134	36	145	30
Left Fronto-central (17)	120	17	143	16	140	15	134	19
Left Central (21)	120	17	129	15	130	16	128	24
Right Frontal (62)	125	33	162	30	141	34	163	27
Right Fronto-central (54)	121	25	154	20	150	25	158	19
Right Central	135	27	144	24	152	27	155	20

### Summary of results for experiment 1

Significant attention-related increases in MMN peak amplitude were observed in adults, and this was more prominent when processing a more difficult to discriminate stimuli. As expected, and consistent with previous studies, the maximal peak was observed in frontocentral regions. However attending to the stimuli also slowed processing speeds; for all three rates a slight delay in processing was observed when comparing the Attend and Ignore conditions. Adults showed a significant lateralization effect, stimuli were processed faster on the left as compared to the right.

## Methods: Effects of Attention and Rate in Auditory Processing in Children

### Participants

Twenty-three monolingual English-speaking children (9 males; mean-age 8 years, 7 months; range 6–11 years) with complete data participated in this study. An additional 8 children were recruited but did not complete the study and thus are not included in these analyses. All children were in their age-appropriate grade in school (mean 3^rd^ grade, SD 1.34) and performed in the normal range on verbal and non-verbal cognitive tasks, had normal hearing, were born full-term and normal birthweight, and had no known neurological or developmental disorders. None reported any family history of language or learning impairments. Rutgers University’s Institutional Review Board approved all study procedures. Written informed consent was obtained from parents or legal guardians of all participating children, and children also provided either written consent or verbal assent for participation.

There were no statistically significant differences between the sample here and the 8 children with incomplete data on demographic or any of the outcome variables assessed [82].

### Procedure

Experiment 2 followed the same protocol as Experiment 1 with the following exceptions: *i) Stimulus probability*. *Based on pilot data that suggested* that this age group had EEGs that were more likely to be contaminated with eye or muscle movement artifacts and to ensure an optimum number of useable trials, children received more deviant trials (166 trials) than adults (125 trials). ii) *Off-line data processing and identifying ERP peaks*. Artifact rejection criteria were set to exclude signals with fluctuations greater than 200 μV. The eMMN was observed between 100–300ms and the LDN was observed between 400–600ms. The above latencies were used as time windows to extract peak amplitude (maximum amplitude) and latency values for the maximum amplitude. Given these differences and the developmental nature of the data in Experiment 2, adult and child samples were not combined for analysis.

### Statistical Analyses

Current analysis paralleled that of Experiment 1: A 4-way Repeated Measures ANOVA (2x2x3x2) was conducted in order to assess the effects of rate (2: *slow*, *fast*), attention (2: *Ignore*, *Attend*), regions of interest (ROI) (3: frontal, frontocentral, and central) and laterality (2: left, right) on the eMMN and LDN peaks. Separate analyses were run for Group I, comparing performance on 300 and 70ms ISIs (G1), and Group II, comparing performance on 70 and 10ms ISIs (G2). Performance on the 300 and 10ms ISIs were compared using independent samples t-tests after verifying that Group 1 (300/70 ms ISIs) and Group 2 (70/10 ms ISIs) did not differ in amplitude (range *t* = 0.38 to 1.38, n.s) or latency for the 70 ms ISI (range *t* = 0.02 to 2.07, n.s). The *F* and *t* statistics ranges presented below are for all 6 channels.

## Results

### Morphology of Children’s Waveform

**Fig 5:**
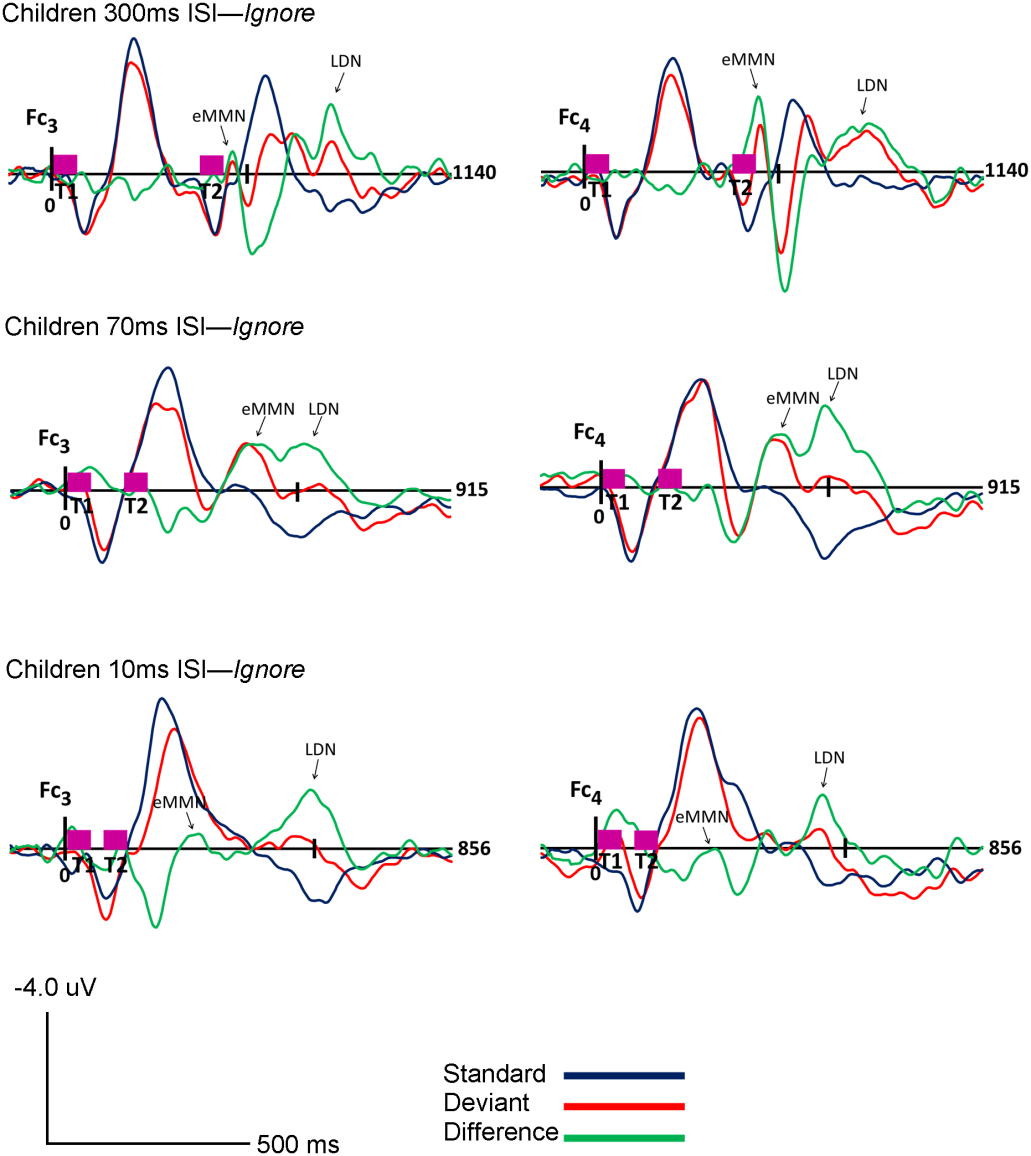
Children *Ignore* Condition. Grand average ERP’s of children for the *Ignore* condition at Fc_3_ (Ch 17) and Fc_4_ (Ch 54) for 300ms (top), 70ms (middle) and 10ms (bottom) ISI’s are shown. Response to the standard stimuli (blue line), the deviant stimuli (red line) and the difference condition (standard- deviant; green line) are plotted. Vertical pink bars on the baseline indicate the onset of the tones. Tone 1 and tone 2 are paired stimuli. Negative voltages are plotted up, positive voltages are plotted down.

**Fig 6:**
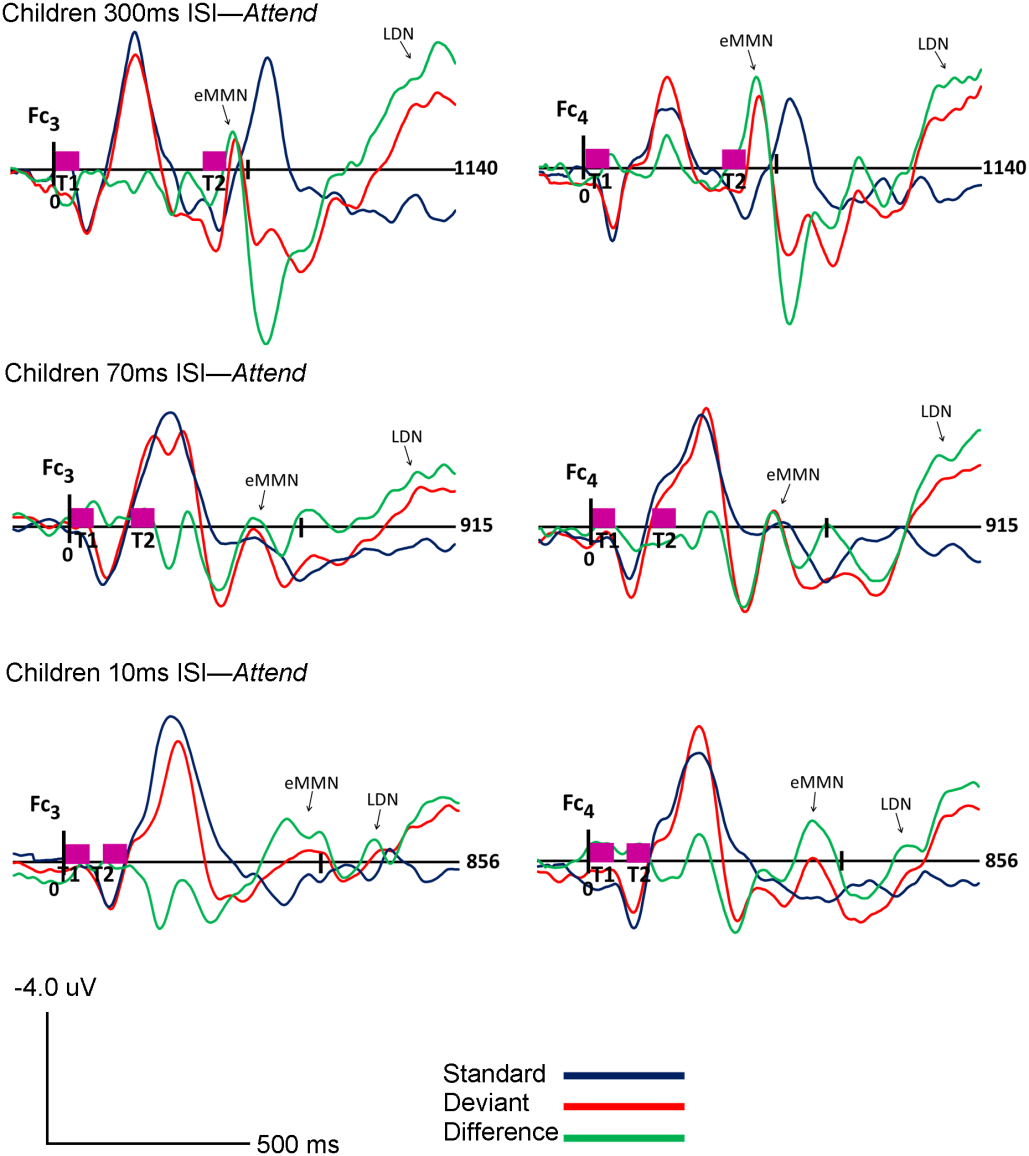
Children *Attend* Condition. Grand average ERP’s of children for the *Attend* condition at Fc_3_ (Ch. 17) and Fc_4_ (Ch. 54) for 300ms (top), 70ms (middle) and 10ms (bottom) ISI’s are shown. Response to the standard stimuli (blue line), the deviant stimuli (red line) and the difference condition (standard- deviant; green line) are plotted. Vertical pink bars on the baseline indicate the onset of the tones. Tone 1 and tone 2 are paired stimuli. Negative voltages are plotted up, positive voltages are plotted down.

**Fig 7:**
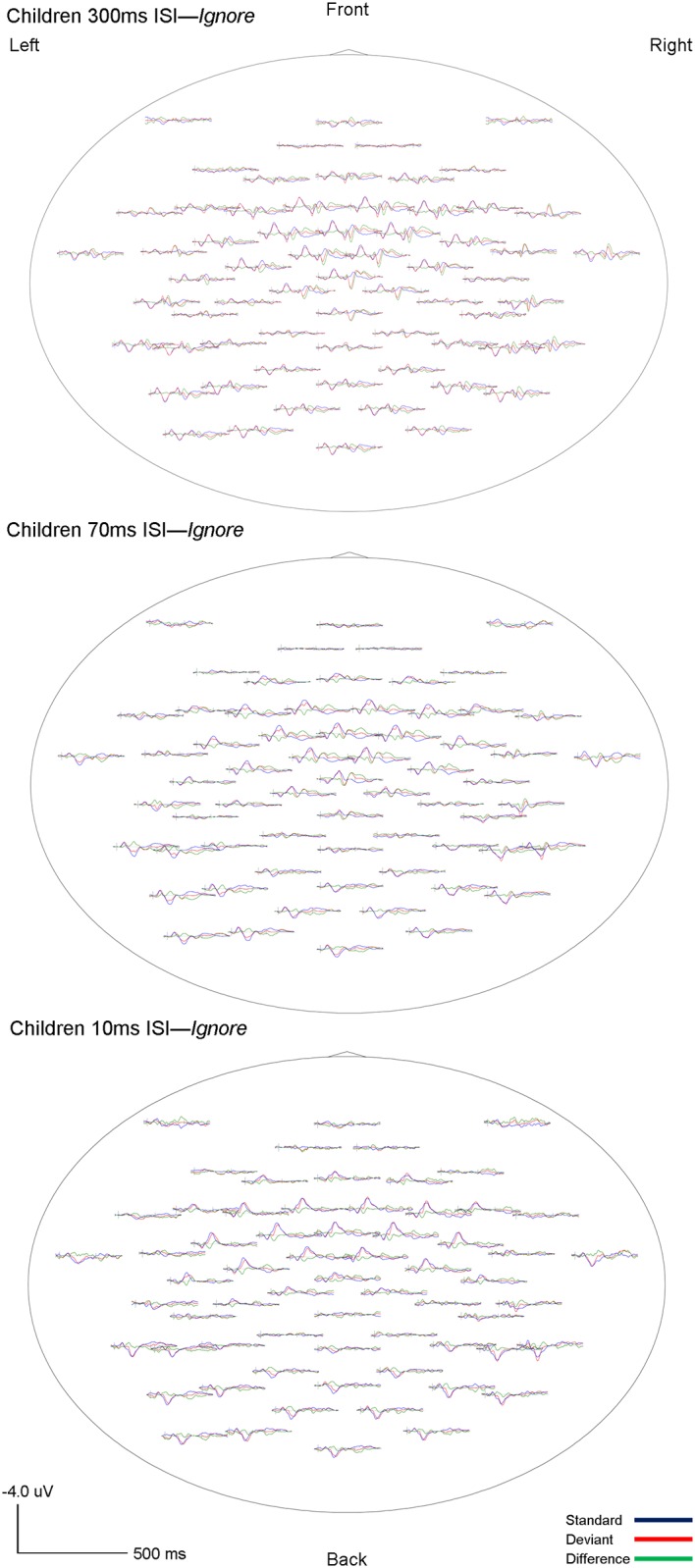
Child *Ignore* grand averaged topographic maps. Whole-head topographies for grand average waveforms for the *Ignore* condition at Fc_3_ (Ch 17) and Fc_4_ (Ch 54) for 300ms (top), 70ms (middle) and 10ms (bottom) ISI’s are shown. Negativity up, positivity down. The standard wave is shown in blue, deviant in red, and difference (deviant-standard) in green. Vertical bars on the baseline indicate the onset of the tones. Tone 1 and tone 2 are paired stimuli.

**Fig 8:**
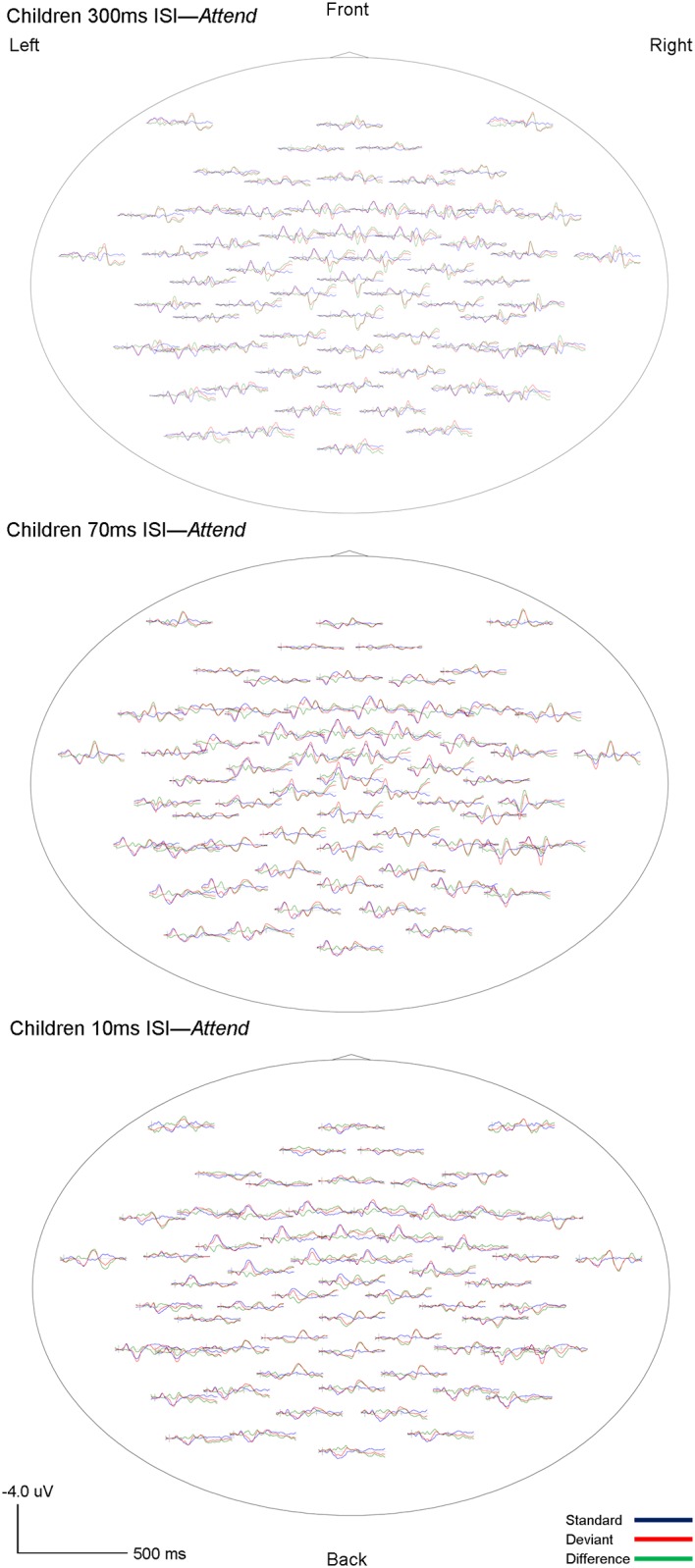
Child *Attend* grand averaged topographic maps. Whole-head topographies for grand average waveforms for the *Attend* condition at Fc_3_ (Ch 17) and Fc_4_ (Ch 54) for 300ms (top), 70ms (middle) and 10ms (bottom) ISI’s are shown. Negativity up, positivity down. The standard wave is shown in blue, deviant in red, and difference (deviant-standard) in green. Vertical bars on the baseline indicate the onset of the tones. Tone 1 and tone 2 are paired stimuli.

The morphology of children’s ERPs was more widely distributed and had higher amplitude fluctuations as compared to adults. However, similar to adults, the highest amplitudes were observed in frontal and frontocentral channels. As expected, two components, eMMN and LDN, were identified in children’s ERP waveforms. Both eMMN and LDN were observed at all three rates of stimuli presentation (300, 70 and 10ms ISI) in both attention conditions (*Ignore* and *Attend)* in all children. The eMMN was broadly distributed but maximal at frontal and frontocentral sites, and associated with large positivities in temporo-mastoid areas. The LDN also had frontal and central topography. The grand-averaged waveforms for children are shown in Figs 5 and 6. Topography of the grand-averaged waveforms for children are presented in Figs 7 and 8.

### Effect of Rate and Attention on Auditory Processing in Children

#### Amplitude Effects for eMMN

Repeated measures ANOVA revealed significant 4-way interactions (Rate x Attention x Region x Hemisphere) for G1 (*F* (1, 4) = 7.59, *p*<0.01) and G2 (*F* (1, 4) = 4.92, *p*>0.05). Further examination revealed an Attention x Hemisphere effect in G1 (*F* (1, 4) = 12.81, *p*<0.05) and G2 (*F* (1, 4) = 6.40, *p*<0.05); the most robust responses were observed for the *Attend* condition in the *Right* hemisphere in both groups. A main effect for Attention was also observed for both groups; in general the *Attend* condition elicited larger peaks compared to the *Ignore* condition (G1, *F* (1, 6) = 51.19, *p*<0.05; G2, *F* (1, 9) = 17.13, *p*<0.05). Rate did not significantly alter the results of these analyses and an independent sample t-tests comparing eMMN peak response for 300 and 10 ms ISI stimuli confirmed that there were no significant rate-related amplitude differences (*t*(21) = 0.01–1.24, *n*.*s*.) (Table 3).

**Table 3:** Means and Standard Deviations of the early Mismatch Negativity (eMMN) Amplitude (mV) in the *Ignore* and *Attend* condition for Children.

	Group I: 300ms vs. 70ms ISI (n = 11)				Group II: 70ms vs. 10ms ISI (n = 12)			
	300ms ISI Mean	SD	70ms ISI Mean	SD	70ms ISI Mean	SD	10ms ISI Mean	SD
*Location (ch)*	**Ignore Condition**							
Left Frontal (13)	-2.20	0.7	-1.56	1.3	-2.23	0.8	-1.02	0.7
Left Fronto-central (17)	-1.89	0.9	-1.52	1.0	-1.52	0.8	-1.05	0.9
Left Central (21)	-1.86	1.5	-0.48	0.9	-1.24	0.9	-0.98	0.8
Right Frontal (62)	-2.05	1.3	-1.15	0.3	-1.61	0.8	-1.22	0.7
Right Fronto-central (54)	-2.25	0.7	-1.71	1.2	-1.95	0.6	-1.02	1.1
Right Central (53)	-1.10	0.7	-0.50	0.7	-1.07	0.9	-1.03	0.8
	**Attend Condition**							
Left Frontal (13)	-2.68	0.4	-2.06	1.3	-2.67	0.8	-2.41	1.9
Left Fronto-central (17)	-2.67	1.5	-2.39	0.2	-2.42	1.2	-1.49	2.0
Left Central (21)	-2.54	1.9	-1.30	1.8	-1.22	1.2	-1.48	1.5
Right Frontal (62)	-3.18	1.3	-2.30	0.9	-2.39	1.3	-2.38	1.9
Right Fronto-central (54)	-3.16	1.1	-2.09	1.1	-2.07	1.5	-1.43	1.5
Right Central (53)	-3.11	1.5	-1.67	1.1	-1.35	1.4	-1.71	1.1

#### Latency Effects for eMMN

A 4-way repeated measures ANOVA revealed a main effect for Rate in G1 only, *300 < 70ms ISI*: (*F* (1, 4) = 31.4, *p*<.00). Independent sample t-test also revealed rate-related differences between 300ms and 10ms ISI peak latencies (300ms < 10ms ISI; *t*(21) = 2.9–4.6, *p*<.05). No differences were found between 70ms and 10ms ISI conditions (Table 4).

**Table 4:** Means and Standard Deviations of the Early Mismatch Negativity (eMMN) Latency (ms) in the *Ignore* and *Attend* condition for Children.

	Group I: 300ms vs. 70ms ISI (n = 11)				Group II: 70ms vs. 10ms ISI (n = 12)			
	300ms ISI Mean	SD	70ms ISI Mean	SD	70ms IS Mean	SD	10ms ISI Mean	SD
*Location (ch)*	**Ignore Condition**							
Left Frontal (13)	153	33	284	33	263	57	280	45
Left Fronto-central (17)	135	12	242	95	256	65	273	23
Left Central (21)	151	26	247	88	251	64	256	21
Right Frontal (62)	143	16	280	25	263	55	287	42
Right Fronto-central (54)	129	4	274	83	268	61	255	27
Right Central (53)	148	21	254	75	263	57	253	44
	**Attend Condition**							
Left Frontal (13)	143	23	268	33	263	66	286	52
Left Fronto-central (17)	136	13	241	92	272	91	287	19
Left Central (21)	154	23	233	23	256	80	286	15
Right Frontal (62)	146	23	239	70	272	52	288	31
Right Fronto-central (54)	131	9	211	77	274	92	281	27
Right Central (53)	142	31	252	32	236	40	279	26

#### Amplitude Effects for LDN

Findings from the LDN analyses differed from the eMMN results. Analyses revealed an Attention x Rate interaction for G1 (*F* (1,4) = 11.34, *p*<0.05) and G2 (*F* (1,4) = 8.54, *p*<0.05), which was decomposed to show that *attending* to the *fast-rate* stimulus (70/10ms ISI) lead to the larger peaks when compared to slower rate stimuli (Table 5).

**Table 5:** Means and Standard Deviations of the Late Difference Negativity (LDN) Amplitude (mV) in the *Ignore* and *Attend* condition for Children.

	Group I: 300ms vs. 70ms ISI (n = 11)				Group II: 70ms vs. 10ms ISI (n = 12)			
	300 ms ISI Mean	SD	70 ms ISI Mean	SD	70 ms ISI Mean	SD	10 ms ISI Mean	SD
*Location (ch)*	**Ignore Condition**							
Left Frontal (13)	-1.47	0.9	-2.15	0.4	-.98	0.5	-1.35	0.5
Left Fronto-central (17)	-1.67	1.2	-1.95	0.7	-1.82	0.4	-1.14	1.0
Left Central (21)	-1.20	1.1	-1.07	0.9	-1.06	0.4	-1.11	0.8
Right Frontal (62)	-2.06	0.8	-1.97	0.6	1.98	0.3	-1.69	0.7
Right Fronto-central (54)	-2.10	1.1	-2.70	0.4	-2.26	0.6	-1.69	0.8
Right Central (53)	-0.99	0.5	-1.67	0.6	-1.20	0.8	-1.45	0.7
	**Attend Condition**							
Left Frontal (13)	-1.70	0.6	-1.64	1.6	-1.70	1.2	-2.60	1.8
Left Fronto-central (17)	-2.10	0.8	-1.63	0.7	-1.72	0.5	-2.14	1.0
Left Central (21)	-1.60	0.8	-1.50	0.7	-1.65	0.6	-1.70	1.0
Right Frontal (62)	-1.70	1.1	-1.98	0.7	-1.75	1.3	-2.38	1.6
Right Fronto-central (54)	-1.48	0.9	-1.51	0.7	-1.50	1.4	-1.30	1.6
Right Central (53)	-1.45	0.5	-1.50	0.7	-1.47	0.9	-2.20	1.2

#### Latency Effects for LDN

Repeated measures ANOVA’s for the latency revealed Rate x Attention interactions for both groups (G1, *F*(1,4) = 10.81, *p*<.05; G2, *F*(1,4) = 7.84, *p*<.05) (Table 6). The latency of the LDN for the more difficult-to-discriminate stimuli (e.g., 70ms and 10ms ISI) in the *Attend* condition was significantly longer than those observed for *Ignore* conditions and for the easier to discriminate *Attend* condition (e.g., 70ms ISI Attend > 300ms ISI Attend, 300 and 70ms ISI Ignore). For G1 there was also a main effect for rate, *300<70ms* ISI (*F* (1,4) = 114.6, *p*<.00), and for G2 there was a main effect for attention (*F* (1,4) = 54.9, *p*<.00), 70ms &10ms ISI Ignore *<* 70 & 10ms ISI Attend).

**Table 6:** Means and Standard Deviations of the Late Difference Negativity (LDN) Latency (ms) in the *Ignore* and *Attend* condition for Children.

	Group I: 300ms vs. 70ms ISI (n = 11)				Group II: 70ms vs. 10ms ISI (n = 12)			
	300 ms ISI Mean	SD	70 ms ISI Mean	SD	70 ms ISI Mean	SD	10 ms ISI Mean	SD
*Location (ch)*	**Ignore Condition**							
Left Frontal (13)	452	44	432	45	441	51	443	43
Left Fronto-central (17)	447	49	440	49	433	60	438	40
Left Central (21)	456	58	425	63	442	60	462	28
Right Frontal (62)	450	26	433	40	446	39	450	49
Right Fronto-central (54)	467	44	435	32	430	32	420	37
Right Central (53)	460	42	440	31	420	41	440	32
	**Attend Condition**							
Left Frontal (13)	466	100	553	66	560	88	573	83
Left Fronto-central (17)	481	51	519	36	537	83	572	67
Left Central (21)	510	96	515	31	528	32	578	43
Right Frontal (62)	478	100	561	46	548	36	580	82
Right Fronto-central (54)	473	58	526	87	521	62	575	24
Right Central (53)	487	88	521	22	540	73	567	42

### Summary of results for experiment 2

When compared to the *Ignore* condition, the *Attend* condition elicited larger amplitude eMMN peaks for all three rates. For the LDN, attention-related amplitude differences were found for the faster-rate stimuli (70ms and 10ms ISI). This is consistent with prior studies suggesting that attending to a stimulus recruits resources at the sensory processing level, but not necessarily at the reorienting or ‘second-look’ phase, especially if the stimulus under consideration is relatively easy to detect and discriminate from others. The latency of the eMMN peak was only affected by rate of presentation, and not attention. For the LDN, however, there was a rate by attention interaction; as expected the more easy-to-discriminate stimuli elicited faster latencies compared to the more difficult to discriminate stimuli. Lateralization effects were observed for the eMMN amplitude only and, in contrast to adult MMN observations, children had larger eMMN’s on the right.

## General Discussion

It is well established that maturational differences in the MMN may be modulated by the type of stimuli used (non-speech vs. speech; simple vs. complex), as well as the perceived difficulty of the discrimination. In both adults and children, the difficulty of the standard-deviant discrimination has been shown to affect the latency and amplitude of the MMN, such that it is characterized by shorter latencies and greater amplitude if the difference between standard and deviant stimuli is large (i.e., stimuli are easily discriminable) ([50, 88–90], but see [31], i.e., for more complex stimuli this relation between latency/amplitude and stimulus difference magnitude may not be monotonic). Little however is known about the role of attention in facilitating this sensory discrimination process and even less is known about the more “upstream”, possibly non-modality specific processes involved in auditory discrimination.

In this study we found that selective attention in adults facilitated acoustic processing resulting in larger MMN peaks. This was particularly true for the more difficult-to-discriminate stimuli (i.e., beginning at the 70ms ISI range). These findings are in line with prior research that suggests that attending to acoustic stimuli initiates the recruitment of additional neural resources or induces greater neural synchrony leading to more robust, albeit slightly later appearing, peaks [73,75]. Children, however, exhibited two separate discrimination peaks (eMMN and LDN). The presence of these peaks were in the expected ranges (100–300, 400–600ms respectively), and, as hypothesized, the independence of these two peaks was demonstrated by their differing responses to changes in rate and attention. These findings are the first to show that the eMMN and LDN differ under different temporal and attentional conditions, and that a more complete understanding of children’s responses to auditory stimuli requires an examination of both peaks. Further, the differences observed here are clearly illustrative of separate cognitive processes and mechanisms underlying these ERPs peaks.

The eMMN in children is thought to be similar in nature to the adult MMN and possibly a precursor of the mature MMN [48, 53]. In these studies we expected, and observed, maturational differences; the eMMN appeared later in time and with larger amplitude when compared to the adult MMN. We also observed significant attention-related amplitude enhancements for all rates in children, and these enhancements were particularly pronounced in the right hemisphere. Adult MMN showed the same pattern of results; that is, there was a main effect for attention and an additional enhancement for the more *difficult-*to-discriminate stimuli. In addition, the adult data showed significant latency effects such that attending to the stimuli resulted in increased latencies of the MMN peak. In children, however, latency differences were only related to presentation rate of the stimuli and, specifically, were longer for the more difficult-to-discriminate rates (i.e. 70 and 10 ms ISI) as compared to the easier 300ms rate. Moreover these effects seem to suggest a threshold-type response as there were no latency differences between the two faster-rate stimuli for the MMN and eMMN. Finally, adults also showed a laterality effect for the MMN (Left > Right), but in children an opposite laterality effect (Right > Left) was observed for the eMMN, but not the LDN, in the attend conditions only.

It is well established that the process indexed by the MMN takes place in the auditory cortex with contributions from frontal regions [22, 33]. The present study supports the hypothesis that children’s processing of complex auditory stimuli may in fact be more reliant on frontal contributions, possibly from anterior cingulate cortex and our findings are in line with the underlying neurodevelopmental changes, specifically ongoing maturation of the frontal generators and the protracted maturational course of prefrontal cortex, which may influence the automaticity of MMN in children as compared to adults [91]. A similar explanation may be used to account for the laterality effect observed only in adults: that is, in a non-disordered adult, consistent and reliable experience with temporally-modulated stimuli may have increased the efficiency of the systems that process these types of stimuli. Given the left hemisphere’s role in resolving brief, rapidly-occurring and successive acoustic cues, such as those found in human speech, it is not surprising that in adults the left hemisphere would be better at generalizing to include these non-speech sounds, thereby demonstrating a hemispheric advantage. In the child this may still be an ongoing process, and more effort may be required to resolve these non-speech sounds that contain linguistic-like acoustic cues.

With regard to the LDN, longer latencies were observed in response to the difficult-to-discriminate rates under attentional control. Given the hypothesis that the LDN is “a second look”, and that the faster rates are in fact more difficult to process, this finding is unsurprising: it should be expected that a developing system takes longer to resolve difficult and more complex stimuli. Further, the LDN is also posited to be dependent on the development of attention mechanisms prior to automaticity [52, 56, 61], thus stimuli that require effortful processing and those that engage selective and focused attention systems, post sensory processing, should lead to longer processing times. Given this explanation, it is possible as well that the attention related latency effect on the LDN observed in children in this study may be influenced by the presence of a P3b-like component typically occurring between the eMMN and LDN. Closer examination of the data does, in fact, suggest that for some of the children there may be a P3b-like component present. This component is not within the scope of this paper and warrants a more complete examination. In relation to the LDN in attend conditions, however, we propose that the presence of the P3b-like peak supports our contention that children are more reliant on active attentional processes in resolving these discriminations as compared to the more automatic processes observed in adults. Interestingly there were no associations between the eMMN and the LDN, suggesting that separate mechanisms may underlie these peaks.

A number of hypotheses have been proposed to account for how attention may function to stimulate change in the auditory cortex. These include either the recruitment of more neural resources during the processing of a stimulus or event [92–95] or an increase in synchrony among neural ensembles responding to the stimuli [96, 97], or both [75, 98]. It has been shown that focusing attention on salient information drives neural dynamics and that selective attention increases both gain and feature selectivity of the mature human auditory cortex [99, 100]. Thus it is likely that either one of these processes may then drive plasticity so that neural responses to sensory events that were initially voluntary become automatic.

An extremely interesting question that unfortunately is outside the scope of this study revolves around the idea that the attention system is key in establishing the memory trace of the standard stimulus (the fidelity of the standard stimuli) to which the deviant stimulus will then be compared [101, 102]. This implies that the MMN functions as a deviance detection device and that the quality of the MMN response will be very dependent upon the fidelity of the standard memory trace. Studies have shown that attention does, in fact, modulate the standard response and not necessarily the deviant one (see [72, 103, 104]). Thus attending to relevant acoustic input may enhance and refine auditory representations established during passive exposure, resulting in greater automaticity of auditory processing and in the short term, may drive plasticity, so that the neural response attains automaticity [105]. However, there is some evidence that top down factors may also modulate initial stimulus-driven auditory organization [102, 103]. It is clear that additional studies are necessary in order to further identify the specific attentional mechanisms and pathways that are engaged in development of acoustic representations.

In sum, the present set of experiments demonstrates that mismatch negativity in adults is influenced by selective attention directed to the auditory modality and modulated as a function of presentation rate. In 6- to 11-year-old children both rate and attention modulation of the two developmental mismatch components were observed, with the eMMN and LDN reflecting somewhat different aspects of auditory discrimination. Thus, in children the eMMN categorically varied as a function of rate, dependent upon whether the stimuli fell inside or outside the developing temporal window of integration, rather than on a “slow to fast” continuum. Moreover, the children’s mismatch components were shown to be differentially influenced by attention in a manner that is consistent with the purported mechanisms they represent. The eMMN reflects early, automatic discrimination and thus attention appears to facilitate processing, while the LDN, purported to index further processing of complex stimuli, is modulated by attention.

Taken together, the present findings contribute to a better understanding of RAP abilities, as indexed by ERP mismatch responses, in mature adults and young school-age children. The results from this set of studies are consistent with the idea that the ability to automatically process subtle auditory changes improves over time due to both maturation and experience-related changes in the brain, and that attention plays a critical part in developing and refining neural auditory processing mechanisms. Given the importance of attention in fine-tuning typically developing auditory processing abilities, one can also posit that atypically developing populations can benefit as well from processing these types of auditory stimuli, which explicitly focuses on engaging the attentional system, thereby normalizing the auditory system.
